# Advanced Microfluidics for Single Cell‐Based Cancer Research

**DOI:** 10.1002/advs.202500975

**Published:** 2025-07-11

**Authors:** Adriana Carneiro, Marta Aranda Palomer, Margarida Esteves, Carolina Rodrigues, José Maria Fernandes, Francisca Oliveira, Alexandra Teixeira, Carlos Honrado, Lorena Dieguez, Sara Abalde‐Cela, Miguel Xavier

**Affiliations:** ^1^ INL ‐ International Iberian Nanotechnology Laboratory Avenida Mestre José Veiga, s/n Braga 4715‐330 Portugal; ^2^ Experimental Pathology and Therapeutics Group Research Centre of IPO Porto / RISE @ CI‐IPOP (Health Research Network) Portuguese Institute of Oncology – Porto Porto Comprehensive Cancer Centre (Porto.CCC) Porto 4200‐072 Portugal; ^3^ Instituto de Ciências Biomédicas Abel Salazar (ICBAS) da Universidade do Porto R. Jorge de Viterbo Ferreira 228 Porto 4050‐313 Portugal

**Keywords:** 3D models, microfluidics, multi‐omics, organ‐on‐chip, single‐cell technologies

## Abstract

Cancer remains one of the leading causes of mortality worldwide, accounting for ≈10 million deaths annually. Critically, it is metastasis and not the primary tumour that causes most of these deaths. Understanding the mechanisms behind cancer dissemination and therapy resistance is thus a pressing challenge. Traditional bulk tissue analyses have failed to capture the full spectrum of intra‐tumour heterogeneity and the dynamic interactions within the tumour microenvironment. Studying cancer at the single‐cell level allows unravelling the roles of rare subpopulations, cell–cell interactions, and spatial dynamics that govern tumour evolution, metastasis, and immune evasion. This review explores how recent advances in microfluidic technologies are transforming ability to model and study cancer at the single‐cell level. Cutting‐edge platforms are highlighted, including droplet microfluidics, single cell‐derived spheroids, and tumour‐chips, that enable physiologically relevant 3D cancer models. By integrating immune components, biosensing, and patient‐derived materials, these platforms hold promise for advancing drug screening, immunotherapy assessment, and personalised medicine. It is concluded by identifying key challenges and priorities for future work, which should focus on increasing model complexity, reproducibility, and integration of spatiotemporal multiomics to better dissect tumour heterogeneity and accelerate clinical translation.

## Graphical abstract

Cutting‐edge microfluidic platforms are transforming single‐cell cancer research. This review highlights advanced technologies, from droplet microfluidics to tumour‐chips, that enable functional and spatial single‐cell analyses. By integrating biosensing, immune components, and patient‐derived materials, these systems offer new insights into tumour heterogeneity and therapeutic responses, paving the way for personalised medicine.

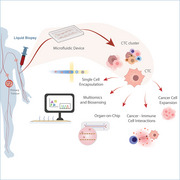

## 1 Introduction

### 1.1 Cancer Facts and Key Figures

Cancer is often perceived as a modern disease, but the first evidence of malignancies dates back thousands of years, from an unmistakable description by the Egyptian physician Imhotep in 2625 BC of ‘a bulging mass in the breast’, to a recently reported 240‐million‐year‐old case of osteosarcoma found in a turtle fossil.^[^ ^1^, ^2^ ^]^ In fact, cancer has been around for much longer than we might be aware. It was Hippocrates, ≈400 BC, who first used a word to describe cancer: *Karkinos*, from the Greek word for “crab”, alluding to the appearance of a tumour with its blood vessels wrapped around it resembling the legs of a crab buried in sand.^[^ ^1^ ^]^ Indeed, cancer only came into the limelight when its burden became hard to ignore. In 2022, cancer accounted for a staggering 9.7 million deaths worldwide,^[^ ^3^ ^]^ which exceeds 1 in every 6 deaths, and the number of new cases is expected to increase by over 50% in less than two decades.^[^ ^3^ ^]^

### 1.2 The Evolution of Cancer Treatment

If cancer mortality suffered astonishing evolution, so has cancer therapy. In the early 20^th^ century, cancer was still viewed as a local disease. In 1882, William Halsted introduced the radical mastectomy, a procedure that involved removing not only the breast but also the surrounding tissues, muscles, and lymph nodes. It reflected the prevailing idea: the more you cut, the better the chances of survival. However, over time, it became evident that such aggressive surgeries were not always curative. Cancer often returned, sometimes in distant parts of the body. The realisation that cancer is not only a local disease, but a systemic one, marked a turning point in our understanding. Treatments began to evolve accordingly, from surgery and radiotherapy to chemotherapy, hormone and targeted therapy, and now immunotherapies. Our view of cancer has transformed radically, but its deadliest hallmark remains: its ability to spread.^[^ ^1^ ^]^

In fact, metastases, rather than the primary tumour, are responsible for the majority of all cancer deaths, with some estimates setting this figure at ≈90% – though this depends on cancer type and patient epidemiology.^[^ ^4^, ^5^ ^]^ Although biologically inefficient, metastasis is enabled by tumour cell evolution, selection, and adaptation. Understanding why certain cells succeed in forming metastases requires looking into cancer evolution, intra‐tumour heterogeneity (ITH), and the role of the tumour microenvironment (TME).

### 1.3 Cancer Evolution and Intra‐Tumour Heterogeneity

Charles Darwin was the first to introduce the concepts of somatic selection, diversity, and extinction. Nearly a century later, Peter Nowell proposed that carcinogenesis is likewise an evolutionary process driven by the selection of genetic alterations that favour survival and proliferation.^[^ ^6^ ^]^ Today, cancer is widely acknowledged as a clonally evolving disease, as cancer cells would not be able to invade, survive, or metastasise, if growth did not occur alongside evolution.^[^ ^1^ ^]^

Each generation of cancer cells introduces genetic diversity.^[^ ^1^ ^]^ Most mutations are either neutral, termed “passenger mutations”, or reduce cell fitness, which leaves newly formed hypermutant cancer cells at risk of being lost by drift or selection.^[^ ^7^ ^]^ Only a few mutations confer a survival advantage. These “driver mutations” are positively selected, and the fittest cancer cells will ultimately survive. Some mutations even promote further mutagenesis.^[^ ^1^ ^]^ This genetic instability, like a perfect madness, only provides more impetus to generate mutant clones.^[^ ^1^ ^]^ This dynamic process results in ITH – the coexistence of genetically distinct cell populations within the same tumour. These subpopulations may differ in their response to therapy or in their potential to metastasise, making ITH a major obstacle to cancer treatment.^[^ ^1^ ^]^

**Figure 1:**
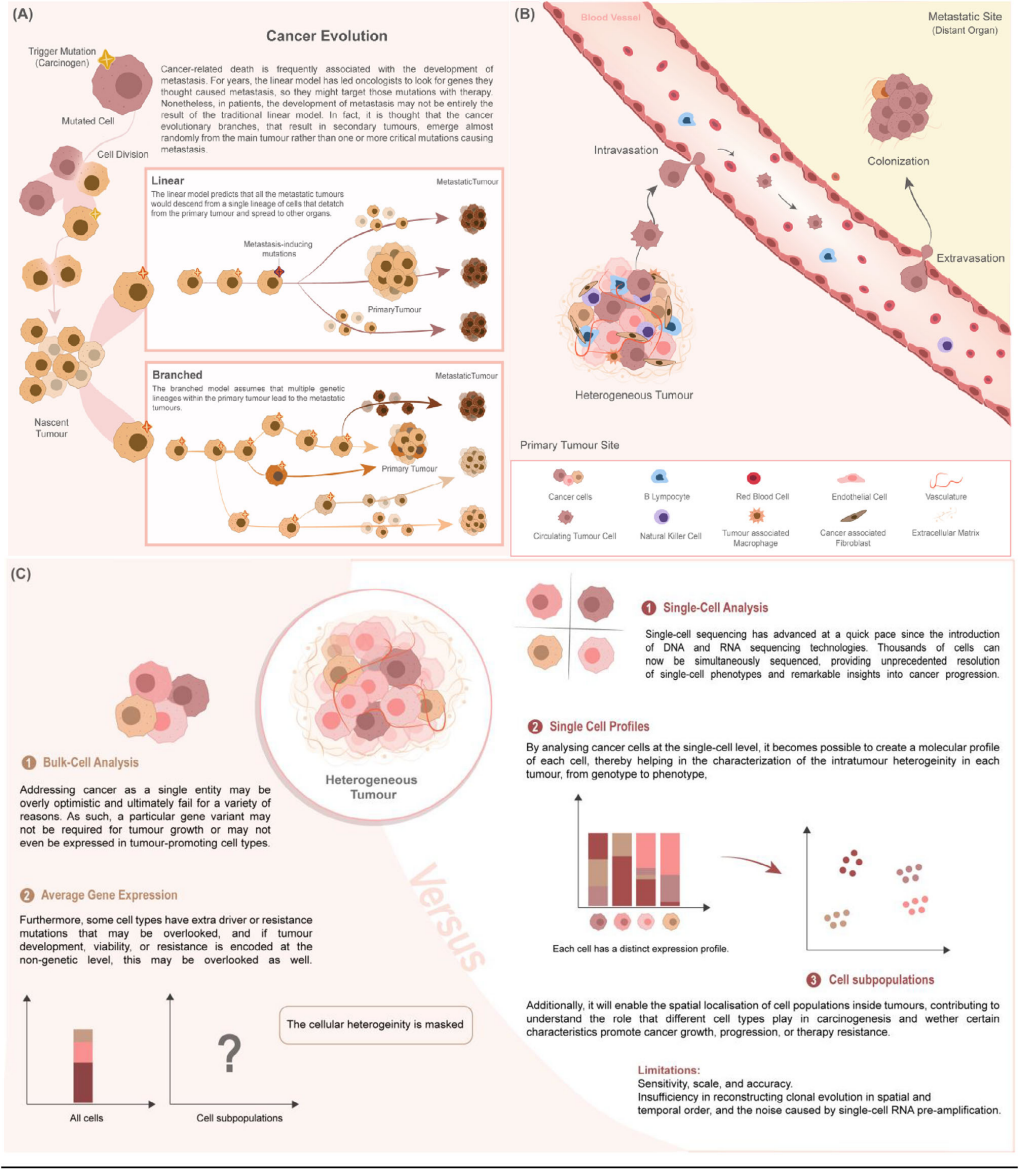
A) Models of cancer evolution. The linear model (on top) supports that each mutation is gradually superseded by the next, resulting in a single, dominant tumour clone. The branched model (on the bottom), where mutations extend and increase like the branches of a tree, demonstrates how different subclones evolve in parallel from a common ancestor, leading to intra‐tumoral heterogeneity with various subclones coexisting within the tumour. B) Tumour heterogeneity, intravasation and extravasation. Schematic representation of intra‐tumoral heterogeneity within the primary tumour, with various sub‐clonal populations. It shows the steps of cancer dissemination, including the detachment of cancer cells from the primary tumour, intravasation into the bloodstream as circulating tumour cells (CTCs), and the subsequent extravasation of CTCs into distant tissues, leading to the formation of metastatic lesions. C) Comparison of bulk and single cell analyses. Bulk analyses provide an averaged molecular profile of the tumour, which can miss critical variations among cells. Single‐cell analyses capture the diversity of individual cells, uncovering heterogeneous gene expression, detecting rare subpopulations, and offering detailed insights into the cellular architecture and functional states within the tumour.

ITH manifests both spatially, across different tumour regions or metastatic sites, and temporally, as tumour subclones evolve over time.^[^ ^7^, ^8^ ^]^ For decades, tumour progression was viewed as a linear process, with each new advantageous driver mutation sequentially replacing previous clones.^[^ ^7^ ^]^ However, advances in genomic profiling and the reconstruction of ancestral relationships of individual subclones have revealed a branched evolutionary model, where multiple subclones emerge in parallel (**Figure** **1**).^[^ ^7^ ^]^ Some cancers may even evolve simultaneously along linear and branched paths. This hybrid model better reflects how tumours adapt to dynamic microenvironments.^[^ ^7^, ^8^, ^9^ ^]^

Understanding cancer evolution and ITH is critical to understanding why some cells succeed in forming metastases while others do not. In addition, It opens new possibilities for personalised cancer medicine by identifying subclonal populations that influence patient prognosis and therapy resistance.^[^ ^7^ ^]^

### 1.4 The Tumour Microenvironment

Cancer has evolved from being thought of as a disease of the cell and its gene expression, to the realisation that it results from a constant, dynamic, and reciprocal interaction between cancer cells and the TME.^[^ ^10^ ^]^ The question then becomes how one can define the TME. A good start would be to think of the TME as a complex and diverse multicellular environment within which a cancer develops. Yet, the TME is only formed after tumour cells colonise normal tissues and alter their surrounding microenvironment – a complicated process that involves, among others, the regulation of immune cells and their secreted factors, and neovascularisation by endothelial cells.^[^ ^11^ ^]^ As a result, the TME includes non‐cancerous cells, such as fibroblasts, endothelial cells, neurons, adipocytes, adaptive and innate immune cells, as well as non‐cellular components such as the extracellular matrix (ECM), and secreted/shed products including cytokines, growth factors, and extracellular vesicles (EVs).^[^ ^11^, ^12^ ^]^

The TME regulates tumour survival and function, potentially allowing cancer cells to develop an invasive phenotype and metastasise.^[^ ^11^ ^]^ Growing evidence reveals that cancer‐associated fibroblasts (CAFs) are essential components that make the TME such a complex and intriguing habitat for cancer cells to evolve. CAFs are the most abundant stromal cells in the TME, and they play a vital role in the majority of processes involved in cancer migration and progression.^[^ ^10^ ^]^ For instance, CAFs are thought to be implicated in tumourigenesis, metastasis, angiogenesis, immune evasion, and drug resistance via cell‐cell interactions. Additionally, they appear to be responsible for the secretion of numerous regulatory molecules and EVs, and particularly, for the synthesis and remodelling of the ECM.^[^ ^10^ ^]^

Our current understanding of the fundamental mechanisms underlying the TME remains limited, and indeed, the presence of CAFs, and other non‐cancer cells in the TME, highlights the diversity of cell phenotypes in a malignant tumour even beyond the heterogeneity of cancer cells. This stresses the importance of studying cancer by dissecting the contributions of each individual player. Bulk analyses may obscure rare but functionally relevant subpopulations, while standard 2D cultures remove the structural and biochemical cues that are essential to tumour behavior. Meaningful progress requires studying individual cells within a context that reflects their interactions with neighbouring cells, the extracellular matrix, and soluble factors (Figure 1).^[^ ^10^, ^11^ ^]^

### 1.5 Looking at Cancer at the Single Cell Level

Sections 1.3 and 1.4 have hopefully conveyed that treating cancer as a single, uniform entity is an oversimplification. For example, certain gene variants may not be essential for tumour growth or may not even be expressed in tumour‐promoting cell types. Additionally, some cells may harbour unique driver or resistance mutations that go undetected in bulk analyses, while non‐genetic factors promoting tumour viability or drug resistance may be similarly overlooked.^[^ ^13^ ^]^ These complexities underscore the need to study cancer using single‐cell methodologies (Figure 1).

Single‐cell sequencing has advanced at a quick pace since the introduction of DNA and RNA sequencing technologies. Thousands of cells can now be sequenced simultaneously, providing unprecedented resolution of single‐cell phenotypes and remarkable insights into cancer progression.^[^ ^14^ ^]^ However, such technologies still face limitations in sensitivity, scale, and spatial resolution. Crucially, they do not fully capture the cellular context. Understanding how a tumour evolves requires more than a list of individual cell states; it requires knowledge of how these cells are spatially arranged and how they interact.^[^ ^14^, ^15^ ^]^

Spatial transcriptomics brings us closer to this goal by mapping gene expression to specific locations within tissues.^[^ ^16^ ^]^ While it is not yet routine to obtain transcriptome‐wide data from all single cells in spatial analyses, the field is advancing rapidly. Soon, we may be able to reach a landmark in cancer research: the capacity to characterize the ITH in individual tumours, from genotype to phenotype, and spatially and temporally map cell populations within tumours using spatiotemporal single‐cell omics in living tissues.^[^ ^17^ ^]^

Equally important is the fact that phenotype is not always a mirror of the genotype, highlighting the importance of multi‐omics analyses. However, for many multi‐omics approaches, sensitivity becomes a limitation at the single‐cell level. For example, the minute quantities of nucleic acids in a single cell require pre‐amplification, which introduces biases in DNA and RNA sequencing.

To overcome this challenge, the appeals of 3D tumour models are two‐fold. First, they enable the expansion of single cells into clonal populations, thereby amplifying biological material to levels suitable for downstream analyses without relying on pre‐amplification. Second, they provide a closer recapitulation of the native tumour microenvironment, preserving critical aspects such as cell‐cell and cell‐matrix interactions and spatial gradients of oxygen, nutrients, and soluble factors.

These 3D cancer models may include cell aggregates, spheroids, organoids, or even patient‐derived xenografts (PDX).^[^ ^19^, ^20^ ^]^ There are many approaches to build 3D models, from scaffold‐based systems that mimic ECM composition to scaffold‐free methods that rely on cell‐driven organisation, including the traditional hanging drop methods, the use of ultra‐low attachment well plates, magnetic levitation, or rotary cell cultures.^[^ ^18^, ^19^ ^]^ More recently, advanced technologies, including 3D‐bioprinting or microfluidics have introduced the capacity to further incorporate complex 3D architectures and/or mechanical stimuli, including shear stress, hydrostatic pressure, or even perfusion.^[^ ^20^, ^21^ ^]^

Despite these advances, most 3D models continue to rely on large starting populations, often hundreds or thousands of cells, which can mask individual cell contributions. Moreover, many studies still use immortalised cell lines that do not reflect the diversity of patient‐derived tumours. To faithfully capture the cellular heterogeneity and dynamic interactions that drive cancer progression, it is essential to move toward systems that both preserve physiological context and are built from primary patient cells. This review focuses on the most recent advancements in the integration of microfluidics and single cell‐based assays as powerful tools for cancer research.

## 2 Sources of Single Cancer Cells

Central to the success of microfluidics‐based cancer research is sourcing relevant cell populations, particularly primary cells from either solid tumour biopsies or liquid biopsies.

### 2.1 Tissue Biopsies

Biopsies remain the gold standard for cancer diagnosis, providing access to primary tumour material for downstream analyses.^[^ ^22^, ^23^ ^]^ For single‐cell studies, tissue samples must be dissociated into viable individual cells, typically through mechanical and enzymatic digestion.^[^ ^24^, ^25^ ^]^ Mechanical dissociation fragments tissue to improve enzyme access, while enzymatic digestion breaks down ECM components and cell‐cell adhesions.

Recent protocols, such as that by Frolova et al.,^[^ ^26^ ^]^ have combined collagenase, hyaluronidase, and DNase I to obtain single‐cell suspensions from triple‐negative breast tumours for scRNA‐seq. Similarly, Yaigoub et al.^[^ ^32^ ^]^ developed a protocol for single cell isolation from human kidney tissue using only 10 mg of biopsy material, by systematically evaluating and combining collagenase types I, II, and IV, with DNase I and hyaluronidase to maximise cell yield and viability. The method included careful control of enzyme concentrations, digestion time, and purification via flow cytometry, and uniquely enabled high‐purity suspensions (>80% viability) from minimal tissue input, making it particularly suitable for clinical applications where tissue availability is limited.

However, isolating pure populations remains challenging due to sample heterogeneity, often requiring additional selection steps.^[^ ^27^, ^28^ ^]^ In addition, tissue biopsies are invasive, limited in frequency, and may not fully capture spatial or temporal tumour heterogeneity.^[^ ^29^, ^30^, ^31^ ^]^ Moreover, as cancer patients are commonly treated with surgical resection of the primary tumour, the tissue itself often becomes unavailable shortly after diagnosis.^[^ ^32^ ^]^ These limitations have motivated increasing interest in alternative sampling strategies, such as liquid biopsies.

### 2.2 Liquid Biopsies

In 1869, Thomas Ashworth identified cells “similar to those in the tumours” in the blood of a metastatic cancer patient.^[^ ^33^ ^]^ Indeed, these cells, which are today ubiquitously known as circulating tumour cells, or CTCs, are infamous for their central role as precursors of metastasis. While the notion that tumour cells circulate freely in the blood of cancer patients may drive a feeling of impending doom, their presence provides unprecedented access to patient cancer cells via a minimally invasive route: the liquid biopsy.

Liquid biopsies provide non‐invasive access to tumour‐derived material, including cell‐free DNA (cfDNA), EVs, and CTCs, via sampling of blood or other body fluids.^[^ ^40^ ^]^ They enable longitudinal monitoring of cancer progression, recurrence, and therapeutic response,^[^ ^36^ ^]^ and several tests have gained clinical approval in recent years.^[^ ^36^ ^]^

CTCs reflect both intra‐ and inter‐tumour heterogeneity.^[^ ^37^ ^]^ However, their isolation is technically demanding due to their scarcity (≈1–10 CTCs per billion blood cells) and lack of a unique biomarker.^[^ ^38^ ^]^ Enrichment strategies exploit biochemical markers (e.g., EpCAM) or biophysical traits (e.g., size, deformability).^[^ ^39^, ^40^ ^]^ While immunoaffinity methods are specific, they may miss mesenchymal or stem‐like subpopulations. On the other hand, label‐free techniques offer broader capture but frequently at lower purity.^[^ ^38^, ^40^, ^41^ ^]^

Recovery of viable CTCs remains a bottleneck for functional studies and 3D culture models.^[^ ^42^, ^43^, ^44^, ^45^ ^]^ Owing to their fragility, short half‐life in circulation, and heterogeneity, successful attempts to establish long‐term cultures of CTCs are rare, but not unprecedented.^[^ ^46^, ^47^, ^48^, ^49^, ^50^, ^51^ ^]^

Zhang et al.^[^ ^52^ ^]^ first reported ex vivo CTC expansion using FACS‐enriched populations from breast cancer patients (based on the expression of CD45, EpCAM and ALDH1). However, in this study, cells were grown in 2D, which has been linked to the loss of important morphological features and functionality, such as cell proliferation and differentiation.^[^ ^49^ ^]^ Yu et al.^[^ ^53^ ^]^ later used the microfluidic CTC‐iChip to isolate CTCs and generate tumour spheres from patients with metastatic breast cancer. The protocol was effective in 6 out of 36 patients, all of whom were not responding to treatment, highlighting how tumour dynamics influence CTC viability. Strategies to increase the success of ex vivo CTC culture include label‐free CTC isolation, hypoxic culture, increasing starting CTC numbers, and co‐cultures with feeder cells such as CAFs.^[^ ^49^ ^]^

### 2.3 Cell Selection Methods

**Figure 2:**
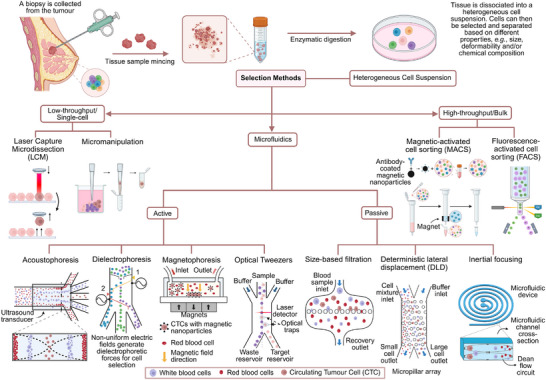
Solid tissue biopsies as a source of single cell suspensions for downstream cancer studies. A cell suspension can be obtained from the solid tissue using a combination of mechanical and enzymatic dissociation processes. The cell suspension is often heterogeneous, and when purity is of concern, a variety of methods can be employed to select for specific cell populations based either on the expression of specific molecular markers or on their biophysical properties. On top of the more conventional manual and bulk selection methods, we focus on microfluidic‐based cell sorting, which is capable of enriching specific cell populations using passive or active sorting. Passive sorting uses hydrodynamic effects generated by the geometry of the microfluidic device, including, as examples, microfiltration, inertial focusing, and deterministic lateral displacement. Active sorting depends on the application of an external force to deflect or trap cells and includes, as examples, acoustophoresis, dielectrophoresis, magnetophoresis, or optical tweezers. Created in BioRender.

Either originating from tissue or liquid biopsies, heterogeneous cell suspensions often require the selection of specific cell populations (**Figure** **2**). For this, a plethora of methods are available and include, but are not limited to, laser capture microdissection (LCM),^[^ ^54^, ^55^ ^]^ micromanipulation or manual cell picking,^[^ ^55^, ^56^ ^]^ and fluorescence‐^[^ ^55^ ^]^ and magnetic^[^ ^57^ ^]^‐activated cell sorting (FACS and MACS). In addition, technologies based on microfluidics have emerged as an alternative offering fast and robust solutions for the isolation of individual cells.^[^ ^58^ ^]^ For a comprehensive review of cell isolation methods, we direct the reader to the review by Zeb et al.^[^ ^59^ ^]^

Briefly, LCM allows the extraction of individual cells or specific populations from tissue samples under microscopic observation. However, it is a complex method, with low throughput, and requires specific skills. Manual cell picking is a simple and efficient method, but like LCM, its throughput is low and requires specialised technicians. Both FACS and MACS depend on the use of antibodies to specific antigens that are present on the cell membrane, cytoplasm, or nucleus.^[^ ^60^ ^]^ In theory, both are able to isolate virtually pure cell populations if appropriate selection markers are available.^[^ ^60^, ^61^ ^]^ However, this is not always the case in cancer samples, and in addition, the use of antibodies can introduce selection biases and compromise cell viability and downstream cell culturing.^[^ ^62^ ^]^

Microfluidics offers the capacity to sort heterogeneous cell populations based on their bio‐physical features. These label‐free approaches are advantageous for their simplicity and for providing unaltered cell populations. Microfluidic sorting can be grouped in two main categories: active sorting and passive sorting. Passive sorting uses hydrodynamic effects generated by the geometry of the microfluidic device, with examples including microfiltration, inertial focusing, or deterministic lateral displacement. Active sorting depends on the application of an external force to deflect or trap cells and include acoustophoresis, dielectrophoresis (DEP), magnetophoresis, or optical tweezers. While active and passive sorting can be used together with antibody labelling to discriminate different cell populations, both are also capable of label‐free sorting using discriminatory features such as cell size and deformability, membrane capacitance (DEP), density (acoustophoresis), or intrinsic magnetic susceptibility (RBCs in magnetophoresis, for example).^[^ ^63^ ^]^

One example is the CTC‐iChip mentioned in the previous section and developed in Toner's lab.^[^ ^64^ ^]^ This device integrates inertial focusing with magnetic deflection to selectively deplete WBCs, thus enabling high‐throughput isolation of viable CTCs from whole blood independently of epithelial marker expression. The chip not only achieves high purity and recovery rates across diverse cancer types, including those with low EpCAM expression or undergoing epithelial‐to‐mesenchymal transition, but also delivers unaltered CTCs compatible with downstream clinical and molecular analyses.

To date, the only FDA‐approved methods for CTC enrichment are the CellSearch CTC test and the Parsortix PC1 system.^[^ ^65^ ^]^ CellSearch, approved for metastatic breast, colorectal, and prostate cancer, separates CTCs using a magnetic field by targeting them with antibody‐labelled ferrofluid nanoparticles, while Parsortix, approved for metastatic breast cancer, captures cells based on their size and deformability.^[^ ^65^ ^]^

The applications of microfluidics for cancer research are significantly more far‐reaching than just cell sorting. Indeed, microfluidics has introduced a plethora of revolutionary technologies that have changed the way we study and understand cancer, as was, for example, the introduction of affordable single‐cell RNA sequencing (scRNA‐Seq) enabled by droplet microfluidics in 2015.^[^ ^66^ ^]^ This manuscript highlights some of these technologies, and we have chosen to focus on droplet microfluidics, organ‐on‐chips, and advanced methods for culturing 3D cell aggregates, which are reviewed in Section 3 and summarised in **Table** **1** at the end of the manuscript.

**Table 1:** Selection of reviewed studies for advanced microfluidic‐based single cell cancer research.

Single Cell‐Derived 3D Spheroids					
Method/Device	Cell Source	Starting Cell Number	Matrix/ECM	Output/Readouts	Reference
DAX‐1 3D cell culture chip (AIM Biotech)	Patient or murine‐derived organotypic tumour spheroids MC38 (murine) & CT26 (human) colon adenocarcinoma cells	Not defined	Type I rat tail collagen	Response to immune checkpoint blockade (PD‐1, CTLA‐4) Live/dead; cytokine profiling (Luminex); RNA sequencing and CIBERSORT	Aref et al.^[^ ^79^ ^]^
Vascularised PDMS microfluidic chip with on‐chip spheroid growth real‐time cell tracking	CTCs derived from colorectal cancer spheroids (HCT116); HUVECs for the endothelium	Single CTCs and CTC clusters were tracked individually	None	Real‐time arrest tracking; metabolic profiling (UPLC‐MS/MS); immunofluorescence (E‐cadherin, N‐cadherin); response to chemotherapy (5‐FU)	Hou et al.^[^ ^80^ ^]^
Dextran drops patterned in PEG reservoirs by density‐adjusted two‐phase aqueous systems	Human (MCF‐7, HepG2, HCT 116, MDA‐MB‐321) and murine (NIH‐3T3, ES D3) cell lines	2500 – 12500 cells per 0.5 µL drops	500 kDa Dextran	Spheroid formation; Differentiation of ES cells Live/dead; Size; RT‐qPCR	Han et al.^[^ ^81^ ^]^
3D spheroid bioprinting using a BioX bioprinter	Human colon adenocarcinoma (SW480, SW620, HCT116, LS174T, HT29, LOVO, CACO2) and rectal adenocarcinoma cell lines (SW1463, SW837)	Printed at 150000 cells mL^−1^ (but drop volume not defined)	Gelatin (10%) / alginate (0.5–5%) bioinks	Drug testing (5‐FU and OX) and radiation‐response Live/dead; Size; Histology (H&E, Safranin O, Sirius Red, Masson's trichrome, HypoxyProbe); Flow cytometry (CD44 and CD133)	Johnson et al.^[^ ^82^ ^]^
Spheroids formed by hanging drop in 2‐µL drops dispensed using I.DOT (Dispendix, DE) Single spheroids printed on µ‐electrode wells for O_2_ sensing using PipeJet® (Biofluidix, DE)	MCF‐7 human breast cancer cells	64 cells per 2 µL drops	None	Response to aerobic metabolism suppressor Antimycin A Live/dead; Size; Oxygen concentration (aerobic metabolism)	Dornhof et al.^[^ ^83^ ^]^
Low attachment plates	Primary epithelial ovarian cancer cells from peritoneal ascites of 9 patients	Single cells	None	2D vs 3D primary cell expansion comparison; Response to carboplatin ICC (PKH67); Flow cytometry (PKH67); Bulk and single cell RNA‐seq; Cell viability (CellTiter‐Glo®)	Velletri et al.^[^ ^84^ ^]^

Droplet Microfluidics					
Method/Device	Cell Source	Starting Cell Number	Matrix/ECM	Output/Readouts	Reference
PDMS device bonded to an electrode‐patterned glass slide. The set‐up included the use of a coded drug library for multiplexing, and lasers and photomultipliers for on‐chip fluorescence detection	U937 human monocytic cells	One cell per droplet	None	Toxicity screen of Mitomycin C Live/dead	Brouzes et al.^[^ ^96^ ^]^
PDMS device where droplets are formed using a capillary‐based liquid handling system	HepG2 human liver cancer cell line	2×10^6^ cells mL^−1^ (but droplet volume not defined)	None	Toxicity screen of Mitomycin C Live/dead	Zhao et al.^[^ ^101^ ^]^
PDMS microdevice for high throughput generation of hydrogel microbeads	HUVECs; Human megakaryoblast leukaemia cells (M07e and MBA2) Mouse embryonic stem cells (R1 and YC5‐YFP‐NEO)	8 × 10^6^ cells mL^−1^	Agarose	Evaluated response to paracrine factors, particularly the secretion of IL‐3 by MBA2 cells on HUVECs	Tumarkin et al.^[^ ^102^ ^]^
PDMS Droplet microfluidic platform including droplet generation and subsequent compartmentalisation into hexagonal anchors individual spheroid cultures	H4‐II‐EC3 rat hepatoma cell line Bovine aorta endothelial cells Human mesenchymal stem cells	1.2 × 10^6^ cells mL^−1^ (≈200 cells per droplet)	0.9% agarose	High‐throughput spheroid generation; Drug testing (acetaminophen) Live/dead; actin organisation; Albumin secretion as a marker of hepatocyte function (ELISA); ICC (Albumin); RT‐qPCR; Drug perfusion	Sart et al.^[^ ^106^ ^]^
Similar to Sart et al. with additional fusion of secondary cell‐laden droplets	Mouse melanoma cell lines (B16 WT, B16‐OVA) and CD8^+^ cytotoxic T lymphocytes (CTLs)	1.5 × 10^6^ cells mL^−1^	2 mg·mL^−1^ Matrigel	Cell‐cell interactions (tumour spheroids and CTLs) Live cell tracking (CTL migration, caspase 3/7 activation, and live/dead)	Ronteix et al.^[^ ^105^ ^]^
Hybrid device incorporating glass, CNC‐machined PMMA, microstructured PDMS, and a PTFE membrane for culturing single cells, cell‐cell pairs, and low cell numbers in hydrogel droplets	HUVECs Human breast cancer cells (MDA‐MB‐231, MDA‐MB‐468, MCF7) Chinese hamster ovary (CHO) cells	3500 cells µL^−1^ with minimum 8‐µL volumes	Agarose	Description of a novel macro‐micro interface for single cell culture and analysis ICC (ALDH1/2, NOTCH1), smRNA FISH (E‐cadherin); RT‐qPCR (CDH1, TOP1)	Kleine‐Brüggeney et al.^[^ ^108^ ^]^
Automated droplet microfluidic platform: PMMA‐based device for droplet generation connected to a 3D‐printed reservoir for rapid production and recovery of spheroids	Human embryonic kidney (HEK293), urinary bladder transitional cell carcinoma (RT4), and epidermoid cancer (A‐431) cell lines	Millions/mL (tens of cells per droplet)	None	Robotically automated spheroid formation and recovery Live/Dead; Spheroid circularity,	Langer& Joensson^[^ ^110^ ^]^
SERS‐microfluidic droplet platform for single‐cell encapsulation and metabolite detection	Human breast (MCF‐7), liver (HepG2), and gastric (SGC) cancer cell lines	Millions/mL (one cell per droplet)		SERS‐based, label‐free, non‐destructive determination of ATP, lactate, and pyruvate from encapsulated single cells	Sun et al.^[^ ^92^ ^]^
SERS‐microfluidic droplet platform (PDMS‐based) for single‐cell phenotypic characterisation	SK‐BR‐3 Human breast (SK‐BR‐3) and melanoma (MDA‐MB‐435) cancer cell lines	Millions/mL (one cell per droplet)		SERS‐based, single cell phenotyping (EpCAM, Vimentin, CD45)	Oliveira et al.

Tumour‐chips					
Method/Device	Cell Source	Starting Cell Number	Matrix/ECM	Output/Readouts	Refs.
Electrical sensing traps in a PDMS microfluidic device (Metas‐Chip)	HUVECs; primary cells from breast tumour and lymph node biopsies (from 70 breast cancer patients) Human breast cancer cell lines (MCF‐7, MDA‐MB‐468)	One HUVEC cell per trap (80 traps) 5/50000 MDA‐MB‐468 Not defined for primary cells	None	Metastatic cell detection via the capacity of cancer cells to displace HUVEC cells in each electrical trap Electrical impedance; H&E; immunohistochemistry (Vim, Pan‐CK); RT‐qPCR (VIM, CDH2, MMP2, MMP9)	Nikshoar et al.^[^ ^128^ ^]^
idenTx (AIM Biotech) comprising a central chamber for endothelial cell and hydrogel loading and lateral channels to drive flow	HUVECs; Fresh patient‐derived glioblastoma cells (from 10 glioblastoma patients) Human glioblastoma cell line U‐87; Patient‐derived glioma stem‐like GS5	∼20000 HUVECs and 5000 glioblastoma cells	2.5 mg·mL^−1^ fibrin	Preferential co‐localisation of single cancer cells with the perivascular niche; and association with PDGFRA expression. Live cell tracking; ICC (Nestin, Sox2, GFAP, Ki67); scRNA‐seq; SEM; 70 kDa Dextran permeability	Xiao et al.^[^ ^10^ ^]^
Custom‐made open‐top PDMS device with interconnected chambers for co‐culture of microvasculature, cancer cells, and macrophages	Normal human lung fibroblasts, Endothelial colony‐forming cell‐derived endothelial cells; PDX‐established CRC and PDAC cell lines CRC (SW480, SW620), PDAC (HPAC, Panc1), and monocyte (THP‐1, U‐937) cell lines	Added at millions/mL (several thousand in total)	10 mg·mL^−1^ fibrin	Effect of macrophage polarisation on tumour growth and tumour‐induced angiogenesis; and its relationship with the production of C‐X‐C chemokines, MMPs, and angiopoietin 2. ICC (vWF, TIMP1, ANGPT2); IHC (CD31 + haematoxylin counterstain); Proteomics (by Olink proteomics); scRNA‐seq	Bi et al.^[^ ^128^ ^]^
Custom‐made PDMS device with two concentric culture chambers for co‐culture of cancer and endothelial cells	HUVECs Bladder tumour cell lines (T24, T24R2)	3200 cancer cells; thousands HUVECs (undefined)	5 mg·mL^−1^ Fibrin; 300 µg·mL^−1^ Matrigel®	Differential response to cisplatin by cisplatin‐resistant/susceptible bladder tumour cells Secretome analysis (cytokine antibody array); RT‐qPCR (*ABCC1, BCL2, CDK6, MCM7, ORC5, SLC31A1, CFLIP*); Cell proliferation (EdU); 70 kDa Dextran permeability	Kim et al.^[^ ^128^ ^]^
Hybrid custom‐made biodevice combining glass, SU‐8 photoresist, electroplated electrodes, PMMA and a PCB for oxygen, glucose and lactate sensing	Breast cancer stem cell line 1 (BCSC1)	3500 to 15000 cells, each forming a single cell‐derived spheroid	75% (V/V) Matrigel®	Response to hypoxia and chemotherapy (Antimycin A, doxorubicin) Glucose, lactate and oxygen sensing	Dornhof et al.^[^ ^128^ ^]^
Custom‐made PDMS device consisting of five parallel channels connected by smaller microchannels or openings for perfusion and co‐culture of cancer and immune cells	Dendritic cells differentiated from CD14^+^ monocytes from PBMCs CRC SW620 cell line	500000 dendritic cells; 25000 SW620	3 mg·mL^−1^ rat tail type I collagen	Evaluation of crosstalk between dendritic cells and cancer cells, succeeding phagocytosis, and relation with the CXCR4 axis Flow cytometry (Annexin V; CD86, CD80, CD123, BDCA3, CD11c, CXCR4); Live cell tracking; Phagocytosis analysis by confocal microscopy; RT‐qPCR (*CCR4, CCL21, CCR2*)	Parlato et al.^[^ ^129^ ^]^
Same device as in Parlato et al.	HUVECs, PBMCs Breast cancer (MCF‐7, BT474) and CAF (Hs578T) cell lines	Cells loaded at 0.25‐5 million/mL Volume not defined but in the few µL range (few thousands of cells)	2.3 mg·mL^−1^ bovine collagen I	Immunocompetent tumour‐chip evaluating the immunomodulation effects of trastuzumab and CAFs Live cell tracking; Flow cytometry (EpCAM, CD31, CD45, CD235a, CD29, FAP, PDGFRb, SMA)	Nguyen et al.^[^ ^130^ ^]^
Custom‐made PDMS device consisting of micro‐well arrays for capturing single cells for the formation of single cell‐derived tumour spheres	Human glioblastoma U251 cells	Cells loaded at 2500 to 25000 per mL to load each chamber with a single cell	None	Evaluation of the relationship between cancer stem cell size and deformability and chemotherapy (vincristine) resistance Live/dead; Cell proliferation (MTT); Mitochondrial membrane potential and Caspase‐3 activation (by fluorescence)	Pang et al.^[^ ^131^ ^]^
Custom‐made PDMS device consisting of integrated droplet generators and microwell arrays for single cell‐laden droplet capture and tumour sphere formation	CRC cell lines (HCT‐116, HT‐29) CRC specimens from 20 patients	Single‐cell derived spheroids	1% (w/v) Alginate	Formation of single cell‐derived spheroids and evaluation of the interdependence between phenotype and adhesion/suspension culture Cell/spheroid size; RNA‐seq	Lin et al.^[^ ^132^ ^]^

5‐FU – 5‐fluorouracil; CAFs – Cancer‐associated fibroblasts; CRC – Colorectal cancer; CTLs – cytotoxic T lymphocytes; ES – Embryonic stem (cells); HUVECs – Human umbilical vein endothelial cells; H&E – Haematoxylin and Eosin; ICC – Immunocytochemistry; IHC – Immunohistochemistry; OX – oxaliplatin; PBMCs – Peripheral blood mononuclear cells; PDAC – Pancreatic ductal adenocarcinoma; PDMS – Poly(dimethylsiloxane); PDGFRA – Platelet‐derived growth factor receptor alpha; PEG – Polyethylene glycol; PMMA – poly(methyl methacrylate); SERS – Surface‐enhanced Raman scattering; UPLC‐MS – Ultra‐performance liquid chromatography‐mass spectrometry.

## 3 Microfluidic Technologies for Single Cell‐Based Cancer Research

At the scale of microfluidics, we enter into the size realm of many interesting biological particles, including proteins, EVs, and animal cells. In addition, in microfluidics, surface effects take on a much bigger role, and viscous forces dominate over inertia. Thus, fluid flow typically occurs in the laminar regime, which makes it predictable and offers outstanding control over its dynamics and over any particles contained within.^[^ ^67^ ^]^ However, when working with reduced volumes and complex samples containing rare events, such as CTCs in whole blood, significant challenges arise, which are summarised in **Table** **2**.

**Table 2:** Challenges and considerations for microfluidic technologies in cancer research.

Cell Source	Throughput
On the one hand, liquid biopsies offer continuous and easy access to a virtually limitless source of cancer cells. On the other hand, the fact that only tumour cells that have acquired the capacity to invade surrounding tissues will be found in circulation introduces a significant bias that may adversely affect studies on tumour heterogeneity. Tissue biopsies, in their turn, are difficult to obtain and preclude longitudinal studies as a regular practice. In addition, they are user‐dependent and can also fail to capture the full heterogeneity of tumours.	CTCs are rare, and the number of CTCs obtained from a typical blood sample can range from a few tens to a few hundred. Likewise, the number of cells obtained from tissue biopsies is limited. Thus, throughput may not be the main concern in microfluidic single cell‐based cancer research. However, when sourcing CTCs from whole blood, removing contaminant blood cells (10^9^ and 10^6^ red and white blood cells respectively) is challenging and requires ultra‐high‐throughput methods.

Reproducibility	Sensitivity
When studying single cells, reproducibility is key. If cells are studied individually, aiming to deconvolute the heterogeneity of the individual parts of bulk samples, each must be studied in the exact same conditions to guarantee that differences originate from cell biology rather than experimental variability. For this, microdroplets can be invaluable for providing well‐defined, monodisperse culture vessels with volumes varying by less than 1%.	There is a wide dynamic range of protein expression in cells, spanning more than six orders of magnitude.^[^ ^68^, ^69^, ^70^ ^]^ Volume scales down as cubic length, and in microfluidics, or in a single 10‐µm diameter cell, picolitre volumes are common. The number of molecules of a protein present in nanomolar concentrations found in one picolitre falls to just a few hundred. And unlike DNA and RNA, metabolites cannot be amplified. Thus, for multi‐omic studies, it is important to work with methods offering enhanced sensitivity.

There is a wide dynamic range of protein expression in cells, spanning more than six orders of magnitude.^[^ ^68^, ^69^, ^70^ ^]^ Volume scales down as cubic length, and in microfluidics, or in a single 10‐µm diameter cell, picolitre volumes are common. The number of molecules of a protein present in nanomolar concentrations found in one picolitre falls to just a few hundred. And unlike DNA and RNA, metabolites cannot be amplified. Thus, for multi‐omic studies, it is important to work with methods offering enhanced sensitivity.

### 3.1 Single Cell‐Derived 3D Spheroids

Solid tumours grow in 3D spatial configurations, and tumour cells are thus unevenly exposed to oxygen and nutrients, as well as other physical and environmental stresses. Accordingly, to comprehensively investigate the pathophysiology of human cancer, it becomes pivotal to be able to preserve or replicate this 3D structure in tissue culture, which has been addressed by a variety of 3D tumour models.^[^ ^71^ ^]^ Specifically, spheroids consist of 3D spherical clusters generated from individual cancer cells, either from immortalised cell lines, primary tumour biopsies, or CTCs, and can recapitulate critical aspects of tumour heterogeneity and the TME.^[^ ^72^ ^]^

In the context of single cell‐based cancer research, these models serve as important downstream platforms for functional studies, especially following the isolation of rare cancer cell populations. While not all studies referenced in this section focus on CTCs, they illustrate the versatility of microfluidic systems for generating spheroids from single cancer cells of diverse origins, supporting drug testing, mechanistic studies, and translational applications.

The process of spheroid formation can be delineated into three stages: i) the creation of loosely connected cell aggregates via integrin‐ECM binding; ii) the upregulation of cadherin expression and its accumulation on the cell membrane; and iii) the development of compact spheroids through cadherin interactions.^[^ ^73^ ^]^ As described in Section 1.5, spheroid cultures can be ECM‐based or ECM‐free. In the former, cells can be either pre‐mixed with uncrosslinked ECM and become embedded upon gelation, or introduced in a pre‐gelled matrix.^[^ ^74^ ^]^ Cells then naturally come together to create spheroids while maintaining matrix binding.^[^ ^75^ ^]^ However, ECM‐free approaches remain the most common, for being simple and cost‐effective.

The hanging drop method uses a small drop of cell suspension that hangs from a surface. The balance between gravity and surface tension converges cells to form an aggregate that eventually evolves to form a spheroid.^[^ ^19^ ^]^ A study by Bahar et al.^[^ ^76^ ^]^ used the hanging drop method to produce spheroids from 1500–2000 ovarian cancer cells to investigate the effectiveness of a combination therapy with niraparib and cisplatin.

The liquid overlay method, where the use of a non‐adhesive surface prevents cell adhesion, forcing cells to cluster up, has also been used to form spheroids.^[^ ^77^ ^]^ In a publication by Khan et al.,^[^ ^80^ ^]^ pancreatic cancer cells were plated in ultra‐low attachment wells to form spheroids, which were subjected to treatment with a mitochondrial metabolism inhibitor (CPI‐613) in combination with Gemcitabine and radiation. The study showed a synergistic effect, and a phase 1 trial to study CPI‐613 in combination with chemo‐radiotherapy was later initiated.^[^ ^78^ ^]^

Over the years, microfluidic devices have also drawn significant attention for the formation of spheroid cultures for their capacity to provide seamless transfer of substances within and around the spheroid using continuous flow.^[^ ^73^ ^]^ In a work by Aref et al., patient‐derived organotypic tumour spheroids were cultured in a commercially available microfluidic device (DAX‐1 from AIM Biotech) to study sensitivity to immune checkpoint blockade therapy using pembrolizumab and ipilimumab. When cultured in collagen hydrogels, the tumour spheroids re‐established native immune cells, including CD8 T cells, which are necessary effectors following treatment with anti‐PD‐L1 antibodies.^[^ ^79^ ^]^

**Figure 3:**
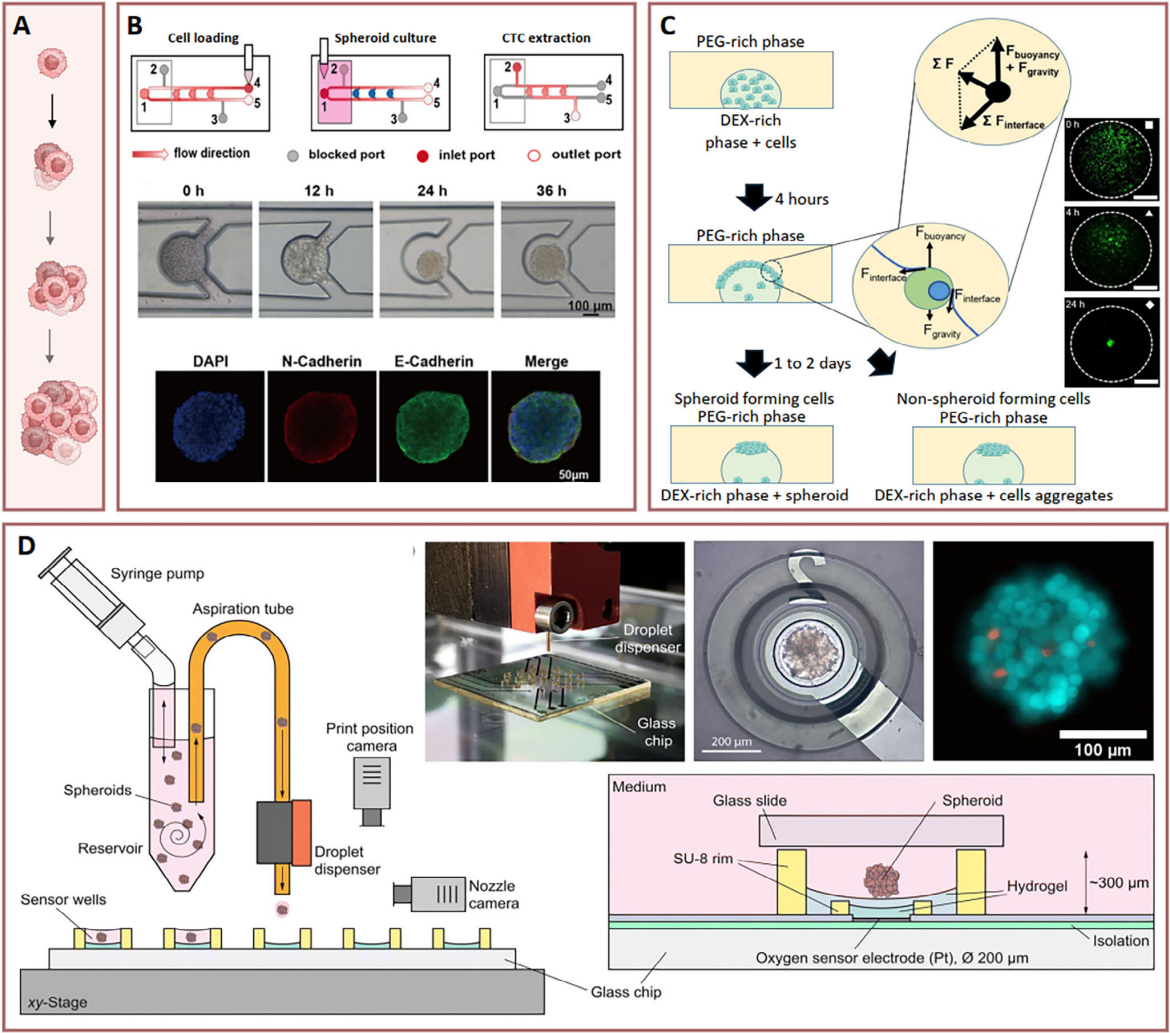
Methods to develop single cell‐derived 3D spheroids. A) Schematic overview of spheroid development starting from a single cell. B) Integrated microfluidic platform for spheroid formation and CTC extraction, as reported by Hou et al.^[^ ^80^ ^]^ The schematic illustrates the chip's multiple functions, including cell loading, on‐chip spheroid culture, and subsequent CTC isolation. Brightfield and fluorescence confocal microscopy were used for spheroid assessment. C) Two‐phase spheroid formation system described by Han et al.^[^ ^81^ ^]^ Cells suspended in the dextran (DEX)‐rich phase migrate upward if less dense than the surrounding phase, accumulating at the interface with polyethylene glycol (PEG). Upon sufficient cell–cell interaction, spheroids form and can be released from the DEX phase and transferred to standard culture plates by introducing PEG/DEX‐free medium. D) Automated spheroid deposition and oxygen monitoring system adapted from Dornhof et al.^[^ ^83^ ^]^ In this setup, pre‐formed spheroids suspended in a reservoir are dispensed into oxygen‐sensing microwells via a droplet‐based bioprinting approach, ensuring uniform distribution and enabling real‐time oxygen measurements.

A particularly innovative technology to connect dynamic cell behavior with downstream omics was presented by Hou et al. (**Figure** **3**A).^[^ ^80^ ^]^ The authors developed a vascularised microfluidic chip to model CTC circulation and arrest under physiologically relevant shear conditions. CTCs, derived from 3D spheroids, were tracked in real‐time to capture their arrest dynamics, and the same single cells were retrieved for MS‐based metabolic profiling. This integrative platform revealed heterogeneity in CTC behavior and metabolism, linking phenotype to function at the single‐cell level.

Despite their potential, microfluidic‐based spheroid formation typically implies the use of complex procedures and equipment and materials that are uncommon in cell culture. Aiming to mitigate these challenges, new methods (microfluidic or not) emerge every day seeking easy‐to‐use and high‐throughput spheroid generation. For instance, Han et al.^[^ ^81^ ^]^ devised an innovative approach that utilises a density‐adjusted polyethylene glycol/dextran aqueous two‐phase system, where cells with a density lower than the dextran‐rich phase float and accumulate at the interface. This allows forming size‐controlled spheroids in a conventional multi‐well plate, and with the potential for high throughput. In addition, by changing the density of the phases, the spheroids can be made to sink, thus switching to surface‐adhesion cultures while negating the need for spheroid manipulation.^[^ ^81^ ^]^

Bioprinting offers another alternative where hydrogel bioinks loaded with cells are deposited with high precision for fast, standardised, and highly scalable spheroid formation. These systems can be further combined with imaging and sensing platforms producing large amounts of data with remarkable spatial and temporal precision as demonstrated by Johnson et al.^[^ ^82^ ^]^ The work by Dornhof et al.,^[^ ^83^ ^]^ further detailed in the **Tumour‐chips** section of this review, is a fine example where 3D bioprinting was combined with a microsensor platform featuring amperometric oxygen sensor electrodes. Using a drop‐on‐demand method, breast cancer spheroids were deposited on sensorised microwells and the metabolic rates of single spheroids when exposed to cancer drugs determined electro‐chemically.

Of outstanding relevance, we highlight the work by Velletri et al.,^[^ ^84^ ^]^ who established 3D spheroid cultures from single cell‐derived metastatic ovarian cancer cells. The authors verified that the spheroids retained key subpopulations from the original patient samples and recapitulated features of the original metastasis that did not emerge in classical 2D cultures. These included both inter‐ and intra‐patient tumour heterogeneity, anticipating their use in patient‐specific drug resistance studies, and to further dissect the mechanisms of metastatic high‐grade serous ovarian cancer.

### 3.2 Droplet Microfluidics

**Figure 4:**
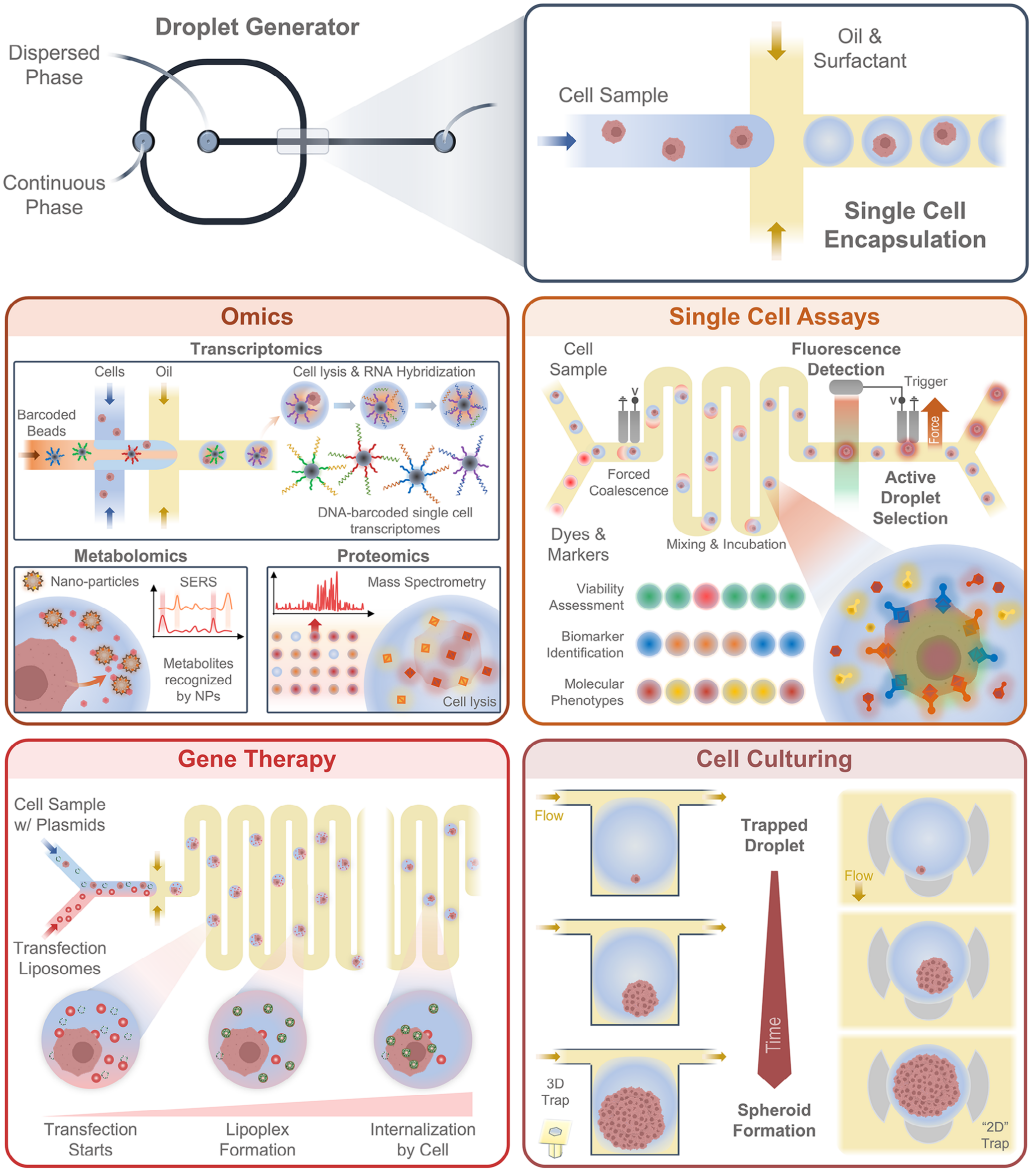
Droplet microfluidics applied to single cell‐based cancer research. Individual cancer cells (or cell clusters) can be encapsulated in individual droplets, generating a self‐contained environment that can be used for a wide range of assays and processes. Multi‐omics analysis of, for example, transcriptome, metabolites, and proteins, can be performed at the single cell level. Individual cells can also be co‐encapsulated with a variety of materials, including dyes/markers for assays to identify viability status or biomarkers/molecules of interest, as well as transfection liposomes for targeted gene therapy on single cancer cells. Encapsulated cells can also be directed to microfabricated trapping features to allow for cell growth and expansion into larger clusters and spheroids.

Microdroplets are minute, liquid structures that result from the emulsification of two immiscible solutions, forming a stable colloidal dispersion, typically within a microfluidic device. Most commonly, they are formed by the segmentation of a water‐based solution, the dispersed phase, by a continuous phase, which is frequently composed of an oil combined with a surfactant^[^ ^85^ ^]^ (**Figure** **4**). In microfluidics, droplets serve as precise vessels for various chemical and biological assays, owing to their throughput in the order of tens of kilohertz, and to pico‐ to nano‐litre volumes with polydispersity in the range of 1%, offering massively parallel and reproducible experimental protocols.^[^ ^85^ ^]^ For example, seminal work by Taly et al.^[^ ^86^ ^]^ introduced a practical, scalable system using droplet microfluidics to perform digital PCR. Since then, the application of droplet‐based digital PCR (ddPCR) has exploded, with different commercial systems being available, and transforming how cancer mutation detection, gene expression quantification, and copy number variation analysis are performed.

In single cell‐based cancer research specifically, droplet microfluidics, together with cell hashing approaches, have revolutionised the field of single‐cell transcriptomics. Since the introduction of single‐cell RNA sequencing (scRNA‐seq) within droplets, the so‐called DropSeq approach, by Macosko et al.,^[^ ^66^ ^]^ further alternatives have been proposed and made commercially available (e.g., Chromium by 10x Genomics^[^ ^87^ ^]^ and BD Rhapsody by Becton Dickinson). This led to a substantial decrease in the cost per cell analysed, enabling massive mapping efforts such as the human cell atlas.^[^ ^66^, ^88^ ^]^

Moreover, the efficient reaction kinetics obtained with the small droplet volumes also make droplet microfluidics an attractive avenue for other “omics” approaches. For proteomics, droplet‐based microfluidics can be integrated with mass spectrometry (MS), opening the possibility for microproteomics, i.e., the study of protein expression in small cell populations or individual cells. By performing in‐droplet sample preparation steps (lysis, digestion, labelling), droplets can then either be merged and extracted before MS injection, or directly electrosprayed onto the MS (using nano‐electrospray ionisation), often after droplet sorting or splitting.^[^ ^89^, ^90^, ^91^ ^]^ This allows for precise, compartmentalised sample handling and minimal contamination or loss before MS analysis.

For metabolomics (and metabolite detection broadly), droplets also provide ideal conditions, as each droplet serves as an independent microreactor, reducing cross‐contamination and improving reproducibility. Single‐cell encapsulation can thus be used to detect cancer biomolecules using, for example, surface‐enhanced Raman scattering (SERS) spectroscopy methods. Sun et al.^[^ ^95^ ^]^ detected the SERS fingerprints of pyruvate, ATP, and lactate, using magnetic nanoparticles co‐encapsulated with breast, cervical, and gastric cancer cell lines. SERS spectroscopy has also been used to detect the expression of membrane surface markers, as was the example of EpCAM in the MDA‐MB‐435 and SKBR3 breast cancer cell lines, co‐encapsulated with gold nanostars in an optofluidic device.^[^ ^93^ ^]^ Despite these applications, single‐cell analysis in cell‐laden droplets can still lack the necessary sensitivity when compared with standard approaches. This is especially relevant for SERS‐based approaches, where detection in the sub‐femtogram level is still limited for metabolites or proteins encapsulated in microdroplets.

The compartmentalisation of single cells within microdroplets also provides the right sterile environment for performing single‐cell assays on live cells.^[^ ^91^, ^94^ ^]^ As an example, Lin et al. developed a sessile microdroplet system for monitoring and analysing cellular interactions between tumour cells and the TME. The results demonstrate that the TME displayed a modulated role in polarising macrophages from an anti‐ tumourigenic to a pro‐tumourigenic phenotype.^[^ ^95^ ^]^ Brouzes et al.^[^ ^96^ ^]^ showed in‐droplet, single‐cell, high‐throughput screening of human monocytic U937 cell viability in droplets, combining a coded drug library. In‐droplet survival rates were high and stable, with the overall cell viability remaining at >80% following incubation for four days. In a work by Yu et al.,^[^ ^97^ ^]^ lymphoma and breast ductal carcinoma cells from spiked blood samples were used to detect matrix metalloproteinases (MMPs) involved in cancer invasion and metastasis. Mazutis et al.^[^ ^98^ ^]^ used droplet microfluidics to detect antibody production in mouse hybridoma 9E10 cells and sort them from the non‐producing human chronic myelogenous leukaemia K562 cells. Single cells were co‐encapsulated together with streptavidin‐coated beads that would capture the secreted antibodies and concentrate the signal of fluorescent detection antibodies.

In‐droplet cell encapsulation can also be a powerful tool to assess gene delivery or gene therapy within individual droplets, aiming, for example, to study the development of mRNA‐based vaccines. In a work by Paris et al.^[^ ^99^ ^]^ A549 lung cancer cells were transfected with a GFP plasmid following encapsulation with lipopolyplexes. Similarly, a study by Li et al.^[^ ^100^ ^]^ showed transfection of K562, THP‐1, and Jurkat cells at efficiencies ranging from 5% to 50% when encapsulated with lipoplexes with the pcDNA3‐EGFP plasmid. The authors hypothesised that the shear stress exerted on cells when passing through the droplet pinch‐off junction increases membrane permeability and subsequent transfection efficiency. The capability of screening vast numbers of encapsulated cells for genomic studies opens the door for large scale studies on individual cancer cells and gene‐based studies, and treatments.

Another major avenue for droplet microfluidics is the development of platforms for single‐cell culture. By encapsulating individual cells in their own isolated microenvironment, the ideal conditions for growing, monitoring, and analysing cells independently are formed. This technique is particularly useful for studying cellular heterogeneity, drug response, and clonal behavior in cancer. In fact, droplet‐based cell culture can be achieved even with simple approaches, without requiring fluid flow in microfluidic devices. One example was presented by Zhao et al.,^[^ ^101^ ^]^ who generated droplets by depositing a cell suspension on the side wall of a PDMS device containing oil, inspired by the hanging‐drop method. This yielded 3D cell cultures in nanolitre‐scale droplets, facilitating in situ observation by confocal microscopy.

Droplets can also be formed using a hydrogel as the dispersed phase, which can promote cell proliferation and provide a 3D environment for cell culture that better approximates in vivo conditions. One example is agarose, which has been shown to allow cell‐cell interactions in microdroplets containing MBA2 and M07e cells.^[^ ^102^ ^]^ Agarose has also been used to encapsulate Jurkat T cells and permit cytokine detection inside droplets (IL‐2, IFN‐g, TNF‐α),^[^ ^103^ ^]^ and to encapsulate human gastric carcinoma cells and breast cancer cells) to detect the expression of the epithelial cell adhesion molecule EpCAM/CD326 by RT‐PCR.^[^ ^104^ ^]^ Hydrogels have also been employed by the Baroud group in microfluidic platforms used to perform high cell‐density and high‐throughput 3D cultures within droplets, with controlled stimuli and while tracking proliferation.^[^ ^105^, ^106^, ^107^ ^]^ In a series of manuscripts, the group demonstrated a system capable of capturing more than 500 individual droplets, which were used to study the generation of spheroids, each from ≈200 cells. Spheroids were formed after a 24‐h incubation period, and their development was tracked individually up to 7 days.^[^ ^106^ ^]^ The platform was also capable of delivering specific cargoes to individual droplets, thus allowing controlled delivery of drugs or chemicals to each spheroid.^[^ ^107^ ^]^

A different system for multiplexed droplet capture and cell proliferation was more recently presented by Kleine‐Brüggeney et al., where traps were positioned in series along a microfluidic device, capturing cell‐laden droplets.^[^ ^108^, ^109^ ^]^ Up to 7700 individual droplets could be trapped, with cell proliferation tracked for up to 80 h. The system also allowed immunostaining and single molecule RNA‐FISH, thus permitting not only tracking cell viability and dynamics but also deciphering their relationship with gene expression. Another system for large‐scale cell studies within droplets was proposed by Langer & Joensson,^[^ ^110^ ^]^ where an inexpensive liquid‐handling robot was programmed to produce scaffold‐free spheroids at high throughput. This effectively allowed the production of 85000 spheroids per microfluidic circuit per hour, while permitting spheroid recovery to standard labware containers and retaining high viability. These strategies for multiplexed formation, manipulation, and analysis of tumour spheroid fate provide vast quantities of spatiotemporally resolved data, thus pinpointing key parameters leading to spheroid formation, and allowing to understand the heterogeneity of tumour outcomes.

Despite the significant advantages, culturing cells in such reduced volumes can be challenging due to nutrient deprivation and the accumulation of cellular waste. For example, in picolitre volumes (a 100‐µm diameter droplet contains 500 pL), the typical millimolar concentrations of glucose quickly amount to just a few picomoles. Note that the glucose consumption of one single cell is in the range of 10^−1^ picomoles per hour.^[^ ^111^, ^112^, ^113^ ^]^ Thus, achieving long‐term cell cultures in microdroplets remains an ongoing challenge. Moreover, the introduction of hydrogels as the dispersed phase can introduce further issues in terms of droplet monodispersity, handling and stability over the long culturing periods. Besides, the application of droplet microfluidics in the context of single‐cell analysis and culture are further limited by issues such as complex setup and operation protocols, gas exchange limitations and material compatibility, limited support for adherent cells, and cell retrieval difficulty.

Alternatively, some of these issues can be circumvented by using microwell chips.^[^ ^114^, ^115^ ^]^ These platforms typically contain hundreds/thousands of individual wells to trap and isolate individual cells. Unlike droplet systems, these wells are usually open structures (in PDMS, glass, or hydrogel) where single cells settle by gravity, or hydrodynamic or electrokinetic sorting. After capture, cells can be locally lysed by thermal or mechanical methods to make RNA, DNA, proteins, and metabolites available for subsequent analysis. They are also widely used for single‐cell culture, high‐resolution single‐cell profiling, imaging, omics analysis, and drug screening, since they provide a stable microenvironment for long‐term observation, are compatible with microscopy and high‐content imaging, and allow recovery and manipulation of individual cells.

Pillai et al.^[^ ^116^ ^]^ developed a PDMS‐based valve‐controlled microfluidic chip platform for the functional profiling of metabolic enzyme activity in individual breast cancer cells, including patient‐derived organoids. Single cells or dissociated PDOs are enzymatically treated, fluorescently tagged, and lysed on‐chip. The authors then use single‐cell activity‐dependent proximity ligation to measure the enzymatic activity of multiple hydrolases. Through this approach, they identified elevated activity of N‐acetylglucosamine‐6‐phosphate deacetylase (NAG6) as a marker of metabolic dormancy. High NAG6 activity was associated with chemoresistance and metastatic potential, establishing it as a functional biomarker for stratifying tumour cells based on drug responsiveness and invasiveness.

### 3.3 Tumour‐Chips

Organ‐chips use microfabricated channels or structures that are specifically designed to host living cells or tissues and replicate the functionality of living organs. They are attractive for allowing highly resolved spatiotemporal tuning of fluid flows, real‐time outflow sampling, the introduction of mechanical cues, and online sensing of multiple parameters like O_2_ and pH.^[^ ^117^, ^118^, ^119^, ^120^ ^]^ Over the past decade, organ‐chips have achieved high physiological relevance and significant clinical mimicry over a wide range of tissues and organs.^[^ ^121^ ^]^ Today, with multiple companies such as Emulate, MIMETAS, TissUse, and others, offering commercially available organ‐chip solutions, one should certainly stop referring to organ‐on‐chip as an emerging technology, but rather as an established technology with a mounting range of applications.

**Figure 5:**
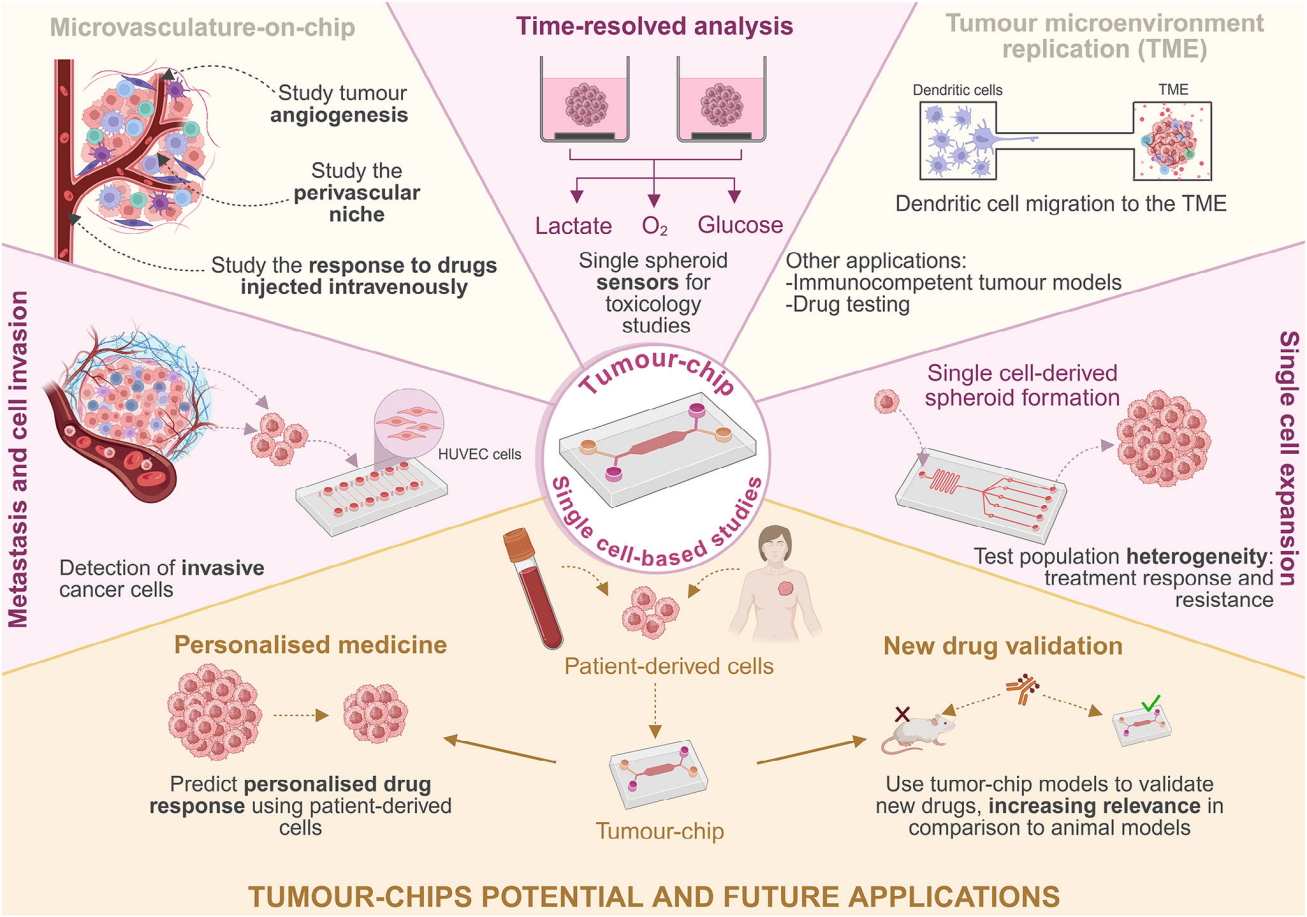
Single cell‐based studies using tumour‐chips. Tumour‐chips can be used to track and characterise single cells, which is crucial to study cancer cell heterogeneity. These models have been used to study invasion and metastasis by tracking cancer cell interaction with single HUVEC cells and thus deriving their invasive capacity. Some tumour‐chip models replicate tumour microvasculature either to study the angiogenic process, the response to drugs injected intravenously, or even to study the perivascular niche through single‐cell scRNA‐Seq, investigating the spatial heterogeneity of the TME. Other models have focused on dissecting tumour cell heterogeneity by expanding single cells into spheroids, which can be crucial to study specific subpopulations that are responsible for treatment resistance. Sensing is essential to track the evolution of 3D structures over time, and there are currently a few studies that have developed single spheroid sensors for O_2_, lactate, and glucose for toxicology studies. Furthermore, the applications of tumour‐chips can be expanded with the introduction of patient derived cells. These models could be used as tools to predict personalised drug responses and they could also be used to test new drugs as an alternative to animal models.

Applications in cancer research are no exception (**Figure** **5**). The first tumour chips precede the year 2010 with examples including devices designed to study drug (doxorubicin) penetration and gradients (nutrients, viable/apoptotic cells, and pH) present in the TME;^[^ ^122^ ^]^ tumour cell migration through a microvasculature endothelial barrier;^[^ ^123^ ^]^ the pharmacokinetics of 5‐fluorouracil (5‐FU) on a multi‐organ‐chip comprising the colon, marrow, and liver;^[^ ^124^ ^]^ and cell‐cell and cell‐ECM interactions affected by autocrine or paracrine signalling.^[^ ^128^ ^]^

However, what is common to these examples is that none simultaneously addressed the importance of mimicking a 3D TME and look at tumour cells at the single cell level. In addition, all used cell lines, from human origin, but none used primary patient cells. Indeed, the field is teeming with examples of significant studies exploiting the capacities of tumour‐on‐chip technology. A quick search on WebofScience using variations of the keywords “tumour‐on‐chip”, “organ‐on‐chip”, and “cancer” returns ≈1000 publications. However, adding the keyword “single cell” reduces this figure to just 103, 31 of which are reviews and 5 are proceedings. It is a selection of these remaining 67 original research publications that will constitute the focus of the next paragraphs.

One example where tumour‐chips have found application for single cell‐based cancer studies is in metastasis and cell invasion. Nikshoar et al.^[^ ^128^ ^]^ used the Metas‐Chip to track cells from liquid biopsies or detached from solid biopsies of breast cancer patients to determine their invasiveness and tendency to retract single HUVEC cells from electrical sensing traps. The device was able to detect invasive cells in 20 tumour samples and 38 lymph nodes of patients with invasive breast cancer, in agreement with H&E, immunohistochemistry, and RT‐PCR analyses. Microvasculature‐on‐chip systems also play a key role in metastasis and cell extra/intravasion studies. Xiao et al.^[^ ^10^ ^]^ found that patient‐derived brain tumour stem cell‐like cells (BTSCs) localise preferentially in the perivascular zone where they show highest motility in their invasive phenotype, migrating over long distances. They further ran scRNA‐Seq to identify tumour cell phenotypes and observed that the co‐localization of BTSCs and microvessels varied greatly between patients and correlated positively with the stem‐like and invasive (mesenchymal) phenotypes. Bi et al.^[^ ^128^ ^]^ also used a vascularised tumour‐chip device to study how M1/M2 tumour‐associated macrophages impacted the TME. The study showed that M2 populations increased tumour cell migration and segregated endothelial cells (as determined by scRNA‐Seq) into distinct subsets of static cells and pro‐angiogenic cells. Kim et al.^[^ ^128^ ^]^ used 3D spheroids from human bladder carcinoma T24 cells in an open‐top vascularised microfluidic device to study resistance to chemotherapy drugs injected intravenously. The open‐top configuration allowed spheroids retrieval and off‐chip scRNA‐Seq analysis. The authors hypothesised that paracrine signalling from the endothelium (HUVECs) might enhance resistance to cisplatin in T24R2 cells.

Sensing in 3D structures becomes paramount to allow time‐resolved analyses in tumour‐chips. Two publications by Dornhof et al.^[^ ^83^, ^128^ ^]^ shows on‐chip culture of 3D spheroids grown from breast cancer cell lines on sensor electrodes for lactate, glucose, and O_2_ sensing. While one work still shows bulk measurements from multiple spheroids,^[^ ^79^ ^]^ the other used bioprinting to place a single spheroid per O_2_ sensor within a 55‐nL volume.^[^ ^83^ ^]^ Although the two publications are mostly engineering demonstrations of the device's robustness and capabilities, the authors demonstrated its potential by showing the real‐time cellular response to antimycin A and doxorubicin, thus underlining significant promise for personalised toxicology studies.

We highlight two studies that use tumour‐chips to replicate the TME. Parlato et al.^[^ ^129^ ^]^ studied dendritic cell migration toward a colorectal cancer cell line following conditioning with IFN‐α. The device, which consisted of large chambers interconnected by microchannels along which dendritic cells could migrate, allowed tracking single dendritic cells as they migrated toward the tumour. When given the option, dendritic cells migrated preferentially toward tumours treated with IFN‐α and the epigenetic drug romidepsin, compared to untreated tumours. Nguyen et al.^[^ ^130^ ^]^ co‐cultured HUVEC cells, primary PBMCs, and cell lines replicating CAFs and breast cancer cells to recapitulate a complex HER2^+^ breast TME and characterise its responses to trastuzumab – a monoclonal antibody specifically used to treat HER2^+^ tumours. The authors observed an antibody‐dependent cell‐mediated cytotoxic response, which was antagonised by CAFs, thus demonstrating an immunocompetent tumour model.

While the following two studies may fall outside the definition of tumour‐chips, they are highlighted here for truly focusing on the power of the single cell. Pang et al.^[^ ^131^ ^]^ reported a microfluidic device that is capable of forming 3D spheroids from single human gliobastoma U251 cells. The device traps single cells in a microarray but uses microfilters to fractionate cells based on cell size and deformability. Single cells are then infused with vincristine, a chemotherapeutic drug. The authors observed that spheroids that originated from smaller/more deformable cells were more resistant to therapy. In a device employing droplet microfluidics, Lin et al.^[^ ^132^ ^]^ were also able to form 3D spheroids originating from single cells. The authors further recovered the chip‐grown spheroids for single‐cell transcriptomic studies but analysed all cells in bulk thus negating the power of single cell‐derived cultures. Nevertheless, they identified differences in the expression of over 1000 genes between the cells grown in adherence and those grown to form single cell‐derived spheroids. Critically, the authors finish by demonstrating the capacity (in 10 out of 20 patients) to grow spheroids starting from single patient‐derived colorectal cancer cells, though at an efficiency lower than 5%.

Finally, a recent study by Ravi et al.^[^ ^133^ ^]^ developed a 3D organotypic breast TME‐on‐a‐chip that integrates patient‐derived CAFs and macrophages with triple‐negative breast cancer cells and couples this system with scRNA‐Seq to reveal their dynamic interplay​. The study showed that the combinatorial presence of CAFs and macrophages significantly enhanced cancer cell invasiveness and proliferation, and triggered immunosuppressive metabolic rewiring by upregulating *KYNU*. Importantly, the use of a small molecule inhibitor targeting this pathway led to reduced tumour cell migration, showcasing the potential for therapeutic screening.

Certainly, tumour‐chips and the capacity to use human cells derived from primary patient samples, in systems which complexity albeit falling short of animal models greatly surpasses that of the classic tissue culture plastic in vitro models, bridge a momentous gap in current cancer research, and their use is bound to continue increasing in years to come offering great hope to the development of personalised treatments. Following the new FDA Modernization Act 2.0 signed in December 2022, new drugs no longer need to be tested in animals to receive approval by the FDA. And indeed, the first clinical trial of a drug which efficacy had only been derived from data obtained in an advanced organ‐chip model has been approved for a drug tackling chronic inflammatory demyelinating polyneuropathy.^[^ ^134^, ^135^ ^]^ Note that this was a drug repurposing, for which safety data still included other models. Nevertheless, there is no doubt that other examples will follow suit in multiple fields of Medicine, including cancer. However, it is important to recognise the limitations of organ‐chip models. While the use of single cells and low cell numbers offers numerous advantages, it also poses challenges as a minimum population size is required to maintain heterogeneity indices in microfluidic assays, as carefully explained in the work by Moore et al.^[^ ^136^ ^]^ In the words of the British statistician George Box, “All models are wrong, some are useful”, and scientists must be alert to what is importantly wrong, thus constantly aiming to choose the model that is best suited to answer the biological question at hand.^[^ ^137^ ^]^

## 4 Outlook

As demonstrated by the examples discussed above, new discoveries in cancer biology, immunobiology, and therapeutic response are being increasingly supported by recent advancements in single‐cell technologies and advanced microfluidic‐based models.^[^ ^138^ ^]^ These models have the potential to transform large‐scale trial testing by providing a more robust and physiologically relevant approach to study tumour complexity compared to traditional 2D cell culture and animal models.^[^ ^139^ ^]^ The combination of microfluidics, advanced 3D bioprinting, and single‐cell profiling technologies facilitates the development of relevant cancer models that closely mimic the cellular, molecular, and functional characteristics of human tumours.^[^ ^140^, ^141^ ^]^ By closely recapitulating the various aspects of the TME, these advanced 3D microfluidic models yield more predictive data for drug screening, unlocking the implementation of personalised therapeutic decisions.

While single‐cell ‐omics have greatly enhanced our ability to decipher the complex cellular and molecular networks within cancer, they remain limited in capturing the full scope of tumour heterogeneity. To address these limitations, advanced experimental platforms, including multiomics techniques, microdroplets, spheroids, and organ‐chip systems using patient‐derived materials, have emerged as some of the most promising tools. For example, microdroplets and single‐cell microfluidics offer high‐throughput, miniaturised, automated, and multiplexed analysis of individual cancer cells. Particularly, microdroplets offer individualised compartments enabling simultaneous examination of diverse cellular parameters, from gene expression profiles to drug responses, within controlled and physiological environments.^[^ ^140^, ^142^ ^]^

The integration of cutting‐edge single‐cell tools with advanced experimental platforms presents a unique opportunity to develop next‐generation 3D cancer models capable of capturing the dynamic interplay between cell types and the surrounding ECM.^[^ ^140^, ^143^ ^]^ Organ‐chips that incorporate these elements have demonstrated significant advantages over conventional 2D cell culture and animal models in recapitulating the pathophysiological, physical, and biochemical cues found in vivo. Additionally, multiomics approaches, which combine various ‘omics data,’ such as genomics, transcriptomics, proteomics, metabolomics, and fragmentomics, have emerged as tools for dissecting tumour heterogeneity and uncovering the mechanisms driving cancer progression and metastasis.^[^ ^144^ ^]^

As such, the development of advanced in vitro models, such as spheroids, organoids, and multi‐organ chips, has further expanded the toolbox for cancer research.^[^ ^144^ ^]^ However, most studies to date have relied on model cell lines, and only a limited number have utilised patient‐derived materials. In tumour‐chips, due to their complexity and low reproducibility, the use of patient‐derived material is almost negligible within the vastness of studies published. Future research should focus on developing more representative and versatile models that incorporate autologous or multi‐patient‐derived cell sources, advanced biosensors, and drug delivery systems.

Hence, the authors´ perspective is that the most promising avenue for future studies is the integration of patient‐derived cells within these innovative microfluidic platforms. In this way, it will be possible to better recapitulate the heterogeneity and complexity of individual patient tumours. This would involve optimising the microfluidic devices to support the culture and analysis of patient‐derived cells, while also incorporating relevant immune components to mimic the TME. These components include tissue samples, organoids, or patient‐derived xenografts. Such integrated models will provide a more clinically relevant platform for studying tumour biology to support the evaluation of therapeutic responses, better reflecting the genetic and phenotypic diversity observed in individual patients.

The inclusion of the immune system within these models will also be crucial for understanding cancer immunobiology and evaluating immunotherapeutic strategies.^[^ ^145^ ^]^ Looking ahead, the development of tumour‐chip systems that integrate autologous or multi‐organ components, as well as the immune system, will be essential to fully capturing the complexity of the TME. Additionally, further optimising these models to enhance physiological relevance, reproducibility, and high‐throughput capabilities will be essential for accelerating the translation of new cancer therapies from laboratory to clinical applications.

Still, studies that originate from single cells are to be kept and pursued, as demonstrated for example, by Lin et al., who used a sessile microdroplet to study pairs of single cells. In a similar approach, Xu et al., profiled the proteomics of immune‐cancer cell interactions using microdroplets technology by leveraging its high‐throughput and precise control of droplet contents.^[^ ^95^ ^]^ Single cancer cell behavior is significantly shaped by interactions with the immune system, impacting tumour progression and therapeutic resistance,^[^ ^146^ ^]^ and immune cells can exhibit both pro‐ and anti‐tumorigenic activities, influenced by the tumour stage.^[^ ^147^ ^]^

The TME, also comprising immune cells, significantly influences tumour development as cancer cells evade the immune system via mechanisms including modulation of PD‐L1 expression.^[^ ^148^ ^]^ Understanding these interactions will improve cancer immunotherapy; and microfluidic platforms are enabling tools for intercellular interaction investigation. Further, future efforts should prioritise the incorporation of further functionalities, such as integrated biosensors and drug delivery systems, to further improve model performance.

In conclusion, optimising these models is crucial to enhance their physiological relevance, reproducibility, and scalability. This will accelerate both our fundamental understanding of cancer and the translation of laboratory findings to clinical applications.

## Conflict of Interest

The authors declare no conflict of interest.

## Author Contributions

S.A.C. and M.X. did conceptualisation. S.A.C., M.X., L.D., C.H., and A.T. did funding acquisition. All authors did the investigation. S.A.C., M.X., L.D., C.H., and A.T. did project administration. S.A.C., M.X., L.D., C.H., and A.T. did supervision. M.X., C.H., A.T., J.M.F., M.A., A.C., M.E., and C.R. did visualisation. M.X. and S.A.C. did Writing – original draft. All authors did Writing – review & editing. All authors revised and approved the final submitted version.
